# Pelvic inflammatory disease associated with cytomegalovirus infection in an immunocompetent adult: Case report and literature review

**DOI:** 10.1002/ccr3.9323

**Published:** 2024-08-11

**Authors:** Yuto Nitta, Takashi Shibata, Hiroki Kato, Satoshi Nakago

**Affiliations:** ^1^ Department of Obstetrics and Gynecology Takatsuki General Hospital Osaka Japan

**Keywords:** abdominal pain, cytomegalovirus infection, immunocompetent adult, infectious mononucleosis, pelvic inflammatory disease

## Abstract

Pelvic inflammatory disease associated with cytomegalovirus infection in immunocompetent adults might be difficult to diagnose because of the rarity and relatively inconspicuous symptoms of infectious mononucleosis. Even if the main complaint is lower abdominal pain, careful search for symptoms latent outside the abdomen could lead to the diagnosis.

## 1 INTRODUCTION

Pelvic inflammatory disease (PID) is an infection of the female genital tract characterized by lower abdominal pain, with the main complaint being genital tenderness.^1^ The causative organisms of PID are known to be chlamydia and gonorrhea, but is clinically considered to be a mixed polymicrobial infection and its precise microbial etiology is unknown in most cases.^2^ We report a case of an immunocompetent adult woman who developed PID symptoms associated with cytomegalovirus (CMV) infection. CMV spreads through body fluids such as blood, saliva, urine, semen, vaginal secretions, feces, or from transplanted organs, and has an affinity for various cells and tissues, resulting in the possibility of infection of any organ throughout the body.^3^, ^4^ Exposure to CMV often occurs in childhood and it persists as an asymptomatic infection throughout life. In some countries, however, CMV seroprevalence among adults has decreased due to sanitary improvements accompanying economic development, and there are an increasing number of reports of adult‐onset infections.^5^ Primary CMV infection is known to cause infectious mononucleosis in immunocompetent adults. However, PID associated with CMV in immunocompetent adults is rare, and the relatively inconspicuous specific symptoms make recognition of CMV infection difficult. In this case study, we present the clinical course and the factors that led to the diagnosis in our patient.

## 2 CASE HISTORY

**FIGURE 1:**
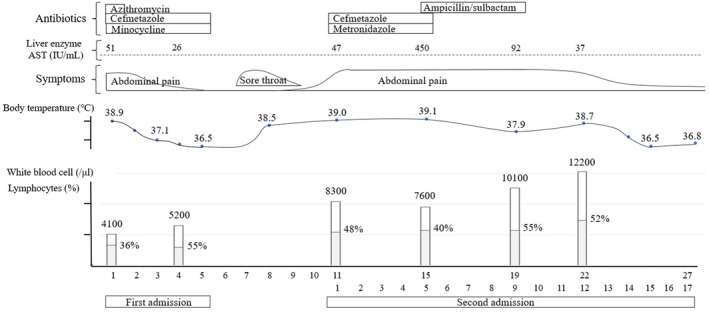
The clinical course of the patient.

A 43‐year‐old nullipara without notable medical history or use of immunosuppressive drugs presented to our hospital with lower abdominal pain and fever (38°C) (Figure 1). White blood cell count was 4800/μL and C‐reactive protein was 4.9 mg/dL (neutrophils 76%, lymphocytes 24%). She was diagnosed with PID based on tenderness in and around the uterus and rebound tenderness in the lower abdomen, and as an inpatient she was administered azithromycin (1 g) once and cefmetazole (4 g), and minocycline (200 mg) daily. The fever and pain resolved, and the patient was discharged on the fifth day. No causative organisms were detected in the blood, urine, or vaginal secretions. Regarding *Chlamydia trachomatis*, the specific IgA‐antigen was positive and IgG‐antigen was positive/negative, but a polymerase chain reaction test of cervical mucus was negative.

Two days after discharge, there was a recurrence of high fever (39°C). The main symptom this time was a sore throat and she visited an internist at another hospital and was diagnosed with the common cold. The patient was prescribed acetaminophen at that time, without antibiotics. However, the fever persisted and 4 days later (6 days after the initial discharge), the patient returned to our facility with a recurrence of lower abdominal pain. By that time, the sore throat had already improved, so her complaint was lower abdominal pain and a 39°C fever. Again, she had lower abdominal tenderness and rebound pain, and an internal examination revealed pain in and around the uterus. With the same symptoms as during her previous hospitalization, the patient was diagnosed with a recurrence of PID.

## 3 METHODS (DIFFERENTIAL DIAGNOSIS, INVESTIGATIONS, AND TREATMENT)

Blood tests on admission showed white blood cells 8300/μL (neutrophils 55%, lymphocytes 45%) and C‐reactive protein 1.3 mg/dL. There were reactive lymphocytes (atypical lymphocytes) of 3%, the upper limit of normal, and a mild elevation of liver enzymes (AST 47 IU/mL, ALT 54 IU/mL). Abdominal ultrasound indicated fatty liver, but we could not identify any abnormalities that could be causing the symptoms. Computed tomography showed no obvious lesions such as an abscess in the pelvis, but there was mild splenomegaly at the upper limit of normal.

Considering the possibility that the duration of the previous antibiotic therapy was insufficient, we started intravenous administration of cefmetazole (4 g/day) and metronidazole (1500 mg/day). On Day 5 of the second hospital admission, there was no improvement of fever and abdominal pain, and liver enzymes had increased (AST 450 IU/mL, ALT 299 IU/mL). We suspected that the antibiotics used were ineffective and might have caused drug‐induced hepatopathy, so we changed the antibiotics to ampicillin/sulbactam (3 g/day). Following this, the liver enzymes showed an apparent improvement to AST 92 IU/mL and ALT 205 IU/mL 4 days after the drug change (Day 9 of the second admission), but there was still no improvement of her symptoms. No causative organisms were detected in the blood, urine, or vaginal secretion cultures. However, inflammation detected by blood tests remained mild compared with the patient's symptoms (white blood cell count up to 8300/μL with lymphocytes predominant up to 55%). Based on the clinical course and laboratory findings, we considered the possibility of a viral infection.

Blood test to confirm a viral infection demonstrated CMV‐specific IgM and IgG antibodies were positive. Epstein–Barr virus, herpes simplex virus, hepatitis B virus, hepatitis A virus, and human immunodeficiency virus were negative and ruled out. Regarding the possibility of CMV infection, the clinical findings outside the lower abdomen were reconfirmed, and there was newly identified lymphadenopathy of the posterior neck. We concluded that this lymphadenopathy and the previously observed findings such as sore throat, splenomegaly of upper normal limit, transiently elevated liver enzymes, and mild reactive lymphocytes (atypical lymphocytes) were the symptoms of infectious mononucleosis associated with CMV infection.

## 4 CONCLUSION AND RESULTS (OUTCOME AND FOLLOW‐UP)

We reckoned that CMV caused the patient's symptoms, so we discontinued the antibiotics on Day 9 of the second admission. CMV DNA was finally detected in the vaginal mucus collected using real‐time polymerase chain reaction method. Her clinical symptoms did not worsen according to observation and they gradually improved. The fever resolved on Day 15 and the abdominal pain disappeared within the following 2 weeks. The patient's clinical course is summarized in Figure 1.

## 5 DISCUSSION

We reported the case of an immunocompetent adult with PID associated with CMV infection who developed lower abdominal pain as the main complaint. Owing to the rarity of PID symptoms developed from CMV and the inconspicuous clinical manifestations of CMV‐induced infectious mononucleosis, it was initially difficult to recognize the causative organism as CMV. When the subjective symptom is pelvic pain and PID is suspected, the focus of examination is likely to be the lower abdomen. However, if symptoms do not respond to antibiotics, considering the possibility of infectious mononucleosis hidden outside the abdomen could lead to a diagnosis.

Primary CMV infection in immunocompetent adults is known to cause infectious mononucleosis. It is mainly caused by the Epstein–Barr virus, but various pathogens including CMV can also cause infectious mononucleosis.^6^ Symptoms include fever, pharyngeal tonsillitis, enlarged cervical lymph nodes, rash, increased peripheral lymphocytes, reactive lymphocytes (atypical lymphocytes), elevation of liver enzymes, and hepatosplenomegaly.^6^, ^7^ In cases of CMV, however, the symptoms are less severe than in cases of Epstein–Barr virus. Lymphadenopathy in the neck and tonsillitis were reported to occur in only 13%–17% of cases of CMV, compared with 80% of cases of Epstein–Barr virus, and splenomegaly is less common in cases with CMV.^3^, ^6^

Regarding female genital infection, to the best of our knowledge, there have been only three reports of CMV infection in immunocompetent adults who developed lower abdominal pain.^8^, ^9^ Only one of these reports described the clinical course^8^; the PID symptoms of fever and pelvic pain and the spontaneous recovery over a number of weeks were similar to those of the patient in our case, but the description of symptoms was entirely focused upon the abdomen. Genital herpes simplex infection was initially suspected in that case due to the abnormal vaginal discharge, vulvodynia, and burning sensation with urination. The diagnosis was based on pathological findings detected from endocervical gland epithelial cells. Most reports of CMV infection that present as abdominal pain were of enteritis in immunocompromised patients or patients using immunosuppressive drugs.^8^, ^9^, ^10^, ^11^ Latent CMV was identified in some previous studies in the genitalia of asymptomatic female adults,^8^, ^12^, ^13^ so the frequency of CMV genital transmission may be underestimated.^12^

Our patient had the same symptoms as the preceding PID, which had improved with antibiotics, so we considered it to be a recurrence. However, the symptoms did not improve, making us aware of the possibility of a viral infection, even after the change in antibiotics. Furthermore, a blood test showed an atypical trend including mild inflammation that contradicted the patient's symptoms and there was an elevated lymphocyte ratio. As a result, CMV‐specific antibodies (IgM and IgG) were confirmed in the blood. Although CMV‐specific antibodies do not necessarily suggest a recent infection, we confirmed clinical findings indicating infectious mononucleosis such as sore throat, lymphadenopathy of the posterior neck, splenomegaly, and reactive lymphocytes (atypical lymphocytes). Elevation of liver enzymes might also be a symptom of infectious mononucleosis, although we suspected drug‐related hepatopathy because the elevation improved after changing the drug. Each symptom was transient and mild, so we considered them to not be directly related to the cause of the lower abdominal pain. The patient recovered spontaneously without treatment, and this clinical course is in line with CMV infection in immunocompetent adults. Finally, CMV DNA was detected in vaginal secretion samples, which led to the diagnosis of PID associated with CMV infection in the female genital tract. As to the possibility that antibiotics might have disrupted bacterial flora and potentially led to the recurrence of CMV, to the best of our knowledge, there have been no reports in immunocompetent patients despite multiple antibiotics being the standard therapy in PID treatment.

In PID, antibiotics often result in symptom relief within 2 weeks, even if the causative organism has not been identified.^14^ When antibiotics are ineffective, it is common to change antibiotics with consideration of drug‐resistant pathogens. In cases of PID in which antimicrobials are ineffective, viral infections including CMV should be considered as differential diagnoses. Even if the main complaint is lower abdominal pain, a reassessment of the systemic physical examination and clinical course to determine the latent symptoms of infectious mononucleosis may lead to an accurate diagnosis.

## AUTHOR CONTRIBUTIONS

**Yuto Nitta:** Writing – original draft. **Takashi Shibata:** Conceptualization; supervision; writing – original draft; writing – review and editing. **Hiroki Kato:** Writing – review and editing. **Satoshi Nakago:** Writing – review and editing.

## FUNDING INFORMATION

No funding was received to assist with the preparation of this manuscript.

## CONFLICT OF INTEREST STATEMENT

The authors declare no conflicts of interest associated with this manuscript.

## CONSENT

Written informed consent was obtained from the patient to publish this report in accordance with the journal's consent policy.

## Data Availability

Data sharing is not applicable to this article as no new data were created or analyzed in this study.
